# Evaluation of dental manifestations in X-linked hypophosphatemia using orthopantomography

**DOI:** 10.1371/journal.pone.0307896

**Published:** 2024-07-26

**Authors:** Rena Okawa, Misato Takagi, Takashi Nakamoto, Naoya Kakimoto, Kazuhiko Nakano

**Affiliations:** 1 Division of Oral Infection and Disease Control, Department of Pediatric Dentistry, Osaka Graduate School of Dentistry, Suita, Osaka, Japan; 2 Department of Oral and Maxillofacial Radiology, Graduate School of Biomedical and Health Sciences, Hiroshima University, Hiroshima, Japan; Texas A&M University College of Dentistry, UNITED STATES OF AMERICA

## Abstract

**Background:**

X-linked hypophosphatemia (XLH) is the most common inherited form of rickets. The presence of sequence variations in the phosphate regulating endopeptidase homolog X-linked (PHEX) gene is associated with increased production of fibroblast growth factor 23 (FGF23). This results in renal phosphate wasting and impaired skeletal mineralization. Spontaneous dental abscesses, caused by endodontic infections resulting from hypomineralization of dentin, are a known dental complication of XLH. There is no objective method to evaluate the severity of dentin dysplasia. The purpose of this study was to develop a quantitative method to evaluate dentin dysplasia using orthopantomography that would allow the values in patients with XLH to be compared with the values in healthy participants of the same age.

**Methods:**

The severity of dentin dysplasia was analyzed by measuring the pulp cavity area of the tooth using orthopantomographic images. The teeth analyzed were mandibular second primary molars and mandibular first permanent molars with complete root formation. Teeth with dental caries, restorations, or root resorption were excluded.

**Results:**

This retrospective observational study included a total of 200 images of healthy participants (aged 2–15 years) divided into five age groups and 42 images of 17 patients with XLH. There was a significant tendency for the pulp cavity area to decrease with increasing age in primary and permanent teeth. The pulp chambers of patients with XLH were larger than those of healthy participants in primary and permanent teeth.

**Conclusion:**

We have established a method of using orthopantomography for quantitative assessment of dentin dysplasia in XLH from the primary dentition to the permanent dentition. Evaluating the severity of dentin hypomineralization by this method is useful in the diagnosis of the dental manifestations of XLH. Early diagnosis of XLH enables oral management and leads to prevention of dental abscesses.

## Introduction

X-linked hypophosphatemia (XLH; OMIM# 307800) is the most prevalent genetic form of rickets. It is characterized by growth retardation, lower limb deformities, and bone and muscle pain [1, 2]. Its prevalence is estimated to be 5.0 per 100,000 [3]. Phosphate regulating endopeptidase homolog X-linked (PHEX) gene sequence variations lead to overproduction of fibroblast growth factor 23 (FGF23), which causes renal phosphate wasting and impaired skeletal mineralization [4].

The lack of phosphate also leads to mineralization defects in teeth [5, 6]. Spontaneous dental abscesses resulting from endodontic infections caused by dentin dysplasia are well known dental manifestations [7]. It is possible for a periapical gingival abscess or fistula to form spontaneously around an apparently healthy tooth, despite the absence of evidence of dental caries or trauma [5–7]. This dental sign may be observed prior to the diagnosis of XLH [8, 9]. Dentin is exposed to oral bacteria through microcracks or attrition of the enamel. The bacteria then invade hypomineralized dentin, causing pulpal necrosis [7]. The pus that is formed by the necrotic pulp drains through the formation of a fistula or dental abscess. There is a no fundamental dental treatment to restore hypomineralized dentin [7]. To prevent pulp infection, it is recommended that early coronal restoration of the teeth be initiated as early as possible, particularly in patients with XLH who are prone to developing repeated dental abscesses [10]. If dentists can objectively diagnose the severity of dentin dysplasia, early prevention of pulp infection is possible.

The manifestations of dentin dysplasia such as a large pulp chamber, thin dentin, and prominent pulp horns are seen radiographically [5, 6, 8]. However, at present, there is no established method for evaluating dentin dysplasia in patients with XLH. If dentists can diagnose dentin dysplasia early, they can provide dental treatment according to the disease severity. Orthopantomography is commonly used in dental practice as a valuable diagnostic tool to fully understand the dental symptoms within the entire maxillofacial region, and is also useful for observing oral and maxillofacial growth and development over time, especially in pediatric dentistry [11]. Studies involving quantitative evaluations with orthopantomography on a millimeter scale for clinical osteoporosis screening have used the mandibular cortical index and the mandibular inferior cortical width below the mental foramen [12–16].

A combination of oral active vitamin D and phosphate salts is the conventional medical therapy of choice for patients with XLH [17]. It has been demonstrated that early intervention with this therapy can result in improvements in the patient’s dental status [18]. However, the treatment cannot completely resolve the dental complications [19]. A humanized monoclonal antibody for FGF23 (burosumab) represents a novel and promising therapeutic approach for XLH [17, 20]. However, the dental effects of this treatment are not yet fully elucidated [7].

The objective of this study was to develop a quantitative method for evaluating dentin dysplasia using orthopantomography, with the aim of enabling comparison between the values of patients with XLH and those of healthy participants.

## Materials and methods

### Collection of orthopantomographic images

This retrospective study used orthopantomographic images stored within the electronic media database of Osaka University Dental Hospital between May and December 2022. All panoramic radiographs were taken with Hyper-X systems (Asahi Roentgen Industries Co. Ltd, Kyoto, Japan). The orthopantomographic images were obtained as part of the dental diagnosis for the treatment of dental caries, periodontal disease, or occlusal problems. One specialist in radiology determined whether the images were affected by movement of the patient and excluded affected images. We collected images from 200 healthy participants without any systemic or chronic disease (American Society of Anesthesiologists [ASA] physical status 1) and divided them into five age groups (2–4, 5–7, 8–10, 11–13, and 14–15 years), with 40 orthopantomographic images in each group. We also collected 42 orthopantomographic images of 17 patients with XLH (8 boys and 9 girls). These images were also taken for diagnosis of dental problems. The 2–4 year age group included eight images from three boys and five girls; the 5–7 year group included 14 images from five boys and nine girls; the 8–10 year group included 10 images from six boys and four girls; and the 11–13 year group included 10 images from three boys and seven girls.

### Medical details of patients with XLH

The following details of patients who visited our clinic for oral management were assessed and recorded: sex, experience of periapical abscess in the primary dentition, medical treatment (conventional therapy or burosumab therapy), age at the time of the first and last examination, and age of taking orthopantomography.

### Methods of evaluation

**Fig 1:**
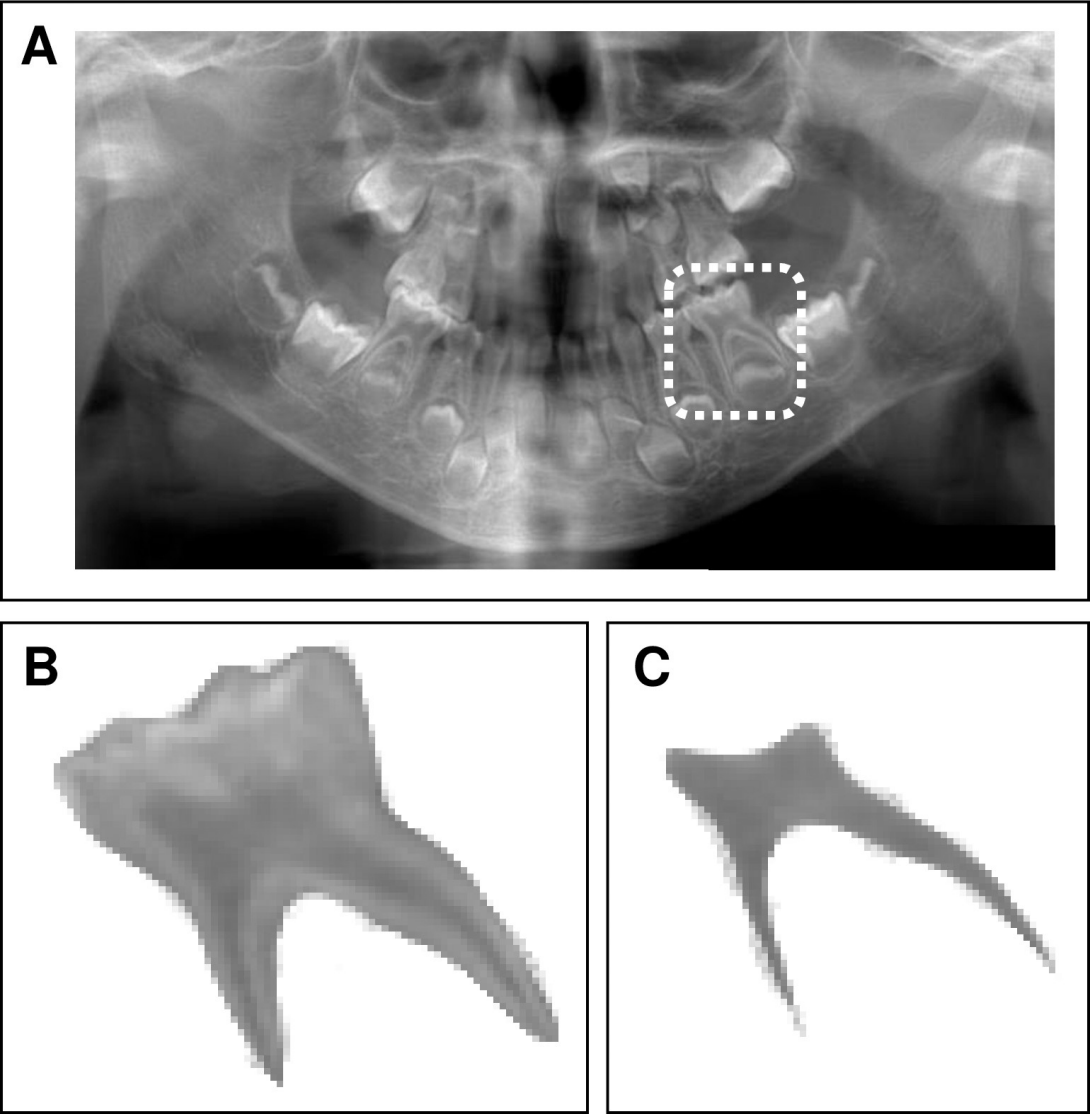
Method for analyzing orthopantomographic images. (A) The target tooth (mandibular left second primary molar) is within the white rectangle. (B) The target tooth trimmed from the orthopantomographic image. (C) The pulp chamber trimmed from the target tooth.

The target teeth for evaluation were bilateral mandibular second primary molars and bilateral mandibular first permanent molars with complete root formation. Teeth with dental caries, restorations, or root resorption were excluded. The target tooth and its pulp cavity were trimmed from the orthopantomographic images by one pediatric dentist (Fig 1). The area of the image obtained using a Java-based image processing program (ImageJ, NIH, Bethesda, MD, USA) was measured, and the percentage of the pulp cavity area in relation to the entire tooth was calculated. To compare the area between treatment methods, three groups were examined: those who had received only conventional therapy, those who had received only burosumab, and those who had transitioned from conventional therapy to burosumab at the time of taking orthopantomography.

### Statistical analysis

Statistical analysis was performed using GraphPad Prism 10 (GraphPad Software Inc., La Jolla, CA, USA). Differences in the incidence of dental abscesses between boys and girls were assessed by Fisher’s exact test. Intergroup differences were compared using analysis of variance. Tukey’s multiple comparison test was used for post-hoc analysis. Trends were analyzed using a linear trend test. The predictive validity of the pulp/tooth ratio results for dentin dysplasia of XLH was assessed using receiver operating characteristic (ROC) curve analysis. The area under the curve (AUC) was assessed for its sensitivity and specificity. The optimal cutoff thresholds for identifying dentin dysplasia with XLH were determined using the highest Youden’s Index. Differences with a *p* value of < 0.05 were considered statistically significant.

### Ethical approval

This study was conducted in accordance with the Declaration of Helsinki (64th World Medical Association General Assembly, Fortaleza, Brazil, 2013) and the Ethical Guidelines for Medical and Health Research Involving Human Subjects. Prior to the commencement of this study, the protocol was reviewed and approved by the Osaka University Graduate School of Dentistry Ethics Committee (approval no. R3-E18). All data collected were fully anonymized prior to their use in this study. The Ethics Committee of Osaka University Graduate School of Dentistry determined that the requirement to obtain informed consent from patients was not necessary. As this was a retrospective observational study using only existing medical records, informed consent was obtained via an opt-out process on the hospital website, which was accessible by all interested parties. Those who elected not to participate in the study were excluded from further involvement.

## Results

### Background of patients with XLH

Orthopantomographic images were obtained from 200 healthy participants (110 boys, 90 girls) and 17 patients with XLH (eight boys, nine girls) from our hospital. Table 1 summarizes details of the patients with XLH in the present study. Six of 17 patients with XLH (35.3%) received conventional therapy, 11.8% (n = 2) received burosumab, and 52.9% (n = 9) transferred from conventional therapy to burosumab (one subsequently switched to conventional therapy). Ten of the 17 patients with XLH (58.8%) (seven boys and three girls) had developed dental abscesses in the primary dentition, and boys were significantly more likely to have a history of a dental abscess (*p* < 0.05).

**Table 1:** Characteristics of patients with X-linked hypophosphatemia (XLH).

Case no.	Sex	Experience of dental abscess	Treatment	Age at dental examination		Age at time of orthopantomography
				First	Last	
1	F	+	C	3Y9M	23Y1M	4Y8M
2	F	-	C	6Y9M	22Y4M	6Y9M
3	M	+	C	7Y0M	23Y6M	11Y6M, 12Y0M
			B (20Y0M~)			
4	M	+	C	9Y8M	23Y2M	9Y8M, 10Y3M
5	M	+	C	8Y8M	10Y5M	10Y3M
6	M	+	C	1Y5M	9Y10M	3Y3M, 4Y4M, 5Y6M, 6Y8M
			B (8Y9M~)			
7	F	-	C	7Y8M	12Y9M	7Y8M, 9Y7M, 11Y0M, 11Y4M, 12Y6M
			B (6Y10M~)			
			C (9Y9M~)			
8	F	-	C	7Y8M	12Y9M	7Y8M, 9Y7M, 11Y4M, 12Y6M
			B (6Y10M~)			
9	M	+	C	7Y8M	12Y1M	7Y8M, 9Y7M, 10Y1M, 11Y1M
			B (11Y1M~)			
10	F	-	C	2Y7M	6Y8M	3Y0M, 4Y1M, 5Y3M, 6Y5M
11	F	+	C	6Y11M	10Y7M	6Y11M, 9Y2M
			B (4Y8M~)			
12	F	-	C	3Y10M	6Y7M	3Y10M, 5Y1M, 6Y1M
			B (3Y2M~)			
13	F	-	C	2Y11M	5Y8M	4Y4M, 5Y5M
			B (2Y4M~)			
14	M	-	B	8Y0M	8Y7M	8Y6M
15	M	+	C	3Y6M	5Y10M	4Y4M, 5Y5M
			B (2Y6M~)			
16	M	+	B	4Y1M	5Y7M	5Y5M
17	F	+	C	9Y10M	12Y3M	9Y10M, 11Y0M, 12Y1M

C, conventional therapy; B, burosumab; C/B, transferred therapy from conventional to burosumab

The age in parentheses in the Treatment column is the age at which the patient switched therapies.

### Pulp/tooth ratio of healthy and XLH participants

**Fig 2:**
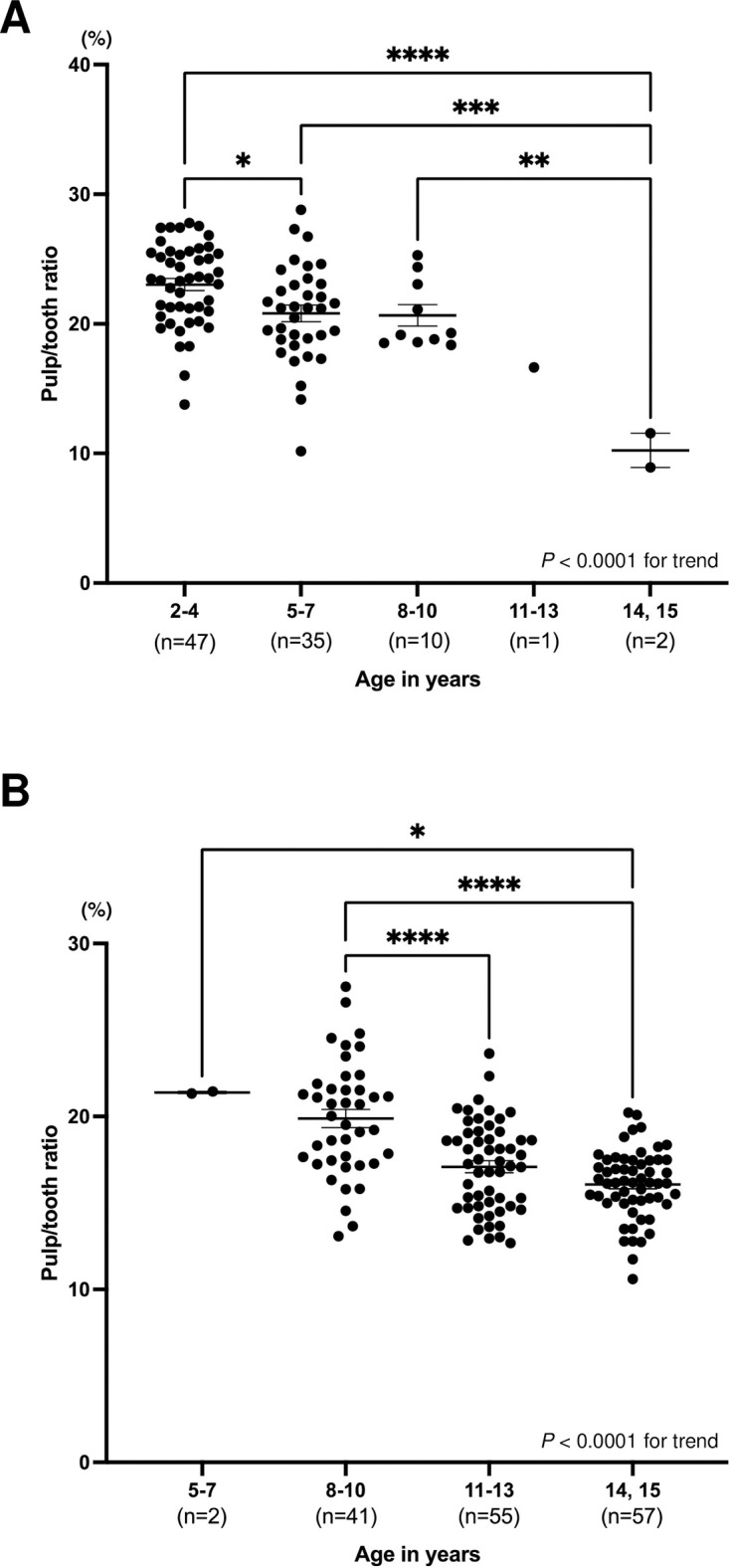
Distribution of the pulp/tooth ratio for each age group in healthy participants. Black horizontal lines in each age group indicate average ± SEM. Significant differences were determined using analysis of variance with Tukey’s multiple comparison test. *p < 0.05, **p < 0.01, ***p < 0.001, and ****p < 0.0001 between groups. (A) Second primary molars. (B) First permanent molars.

The number of analyzed teeth in healthy and XLH participants is shown in Table 2. There was a significant tendency for the pulp/tooth ratio to decrease with increasing age in primary and permanent teeth (*p* < 0.001) in healthy participants (Fig 2). The pulp/tooth ratio of primary teeth in healthy participants was 23.0 ± 0.5 (average ± SEM) in the 2–4 year group (n = 47), 20.8 ± 0.6 in the 5–7 year group (n = 35), 20.7 ± 0.8 in the 8–10 year group (n = 10), 16.6 in the 11–13 year group (n = 1), and 10.2 ± 1.3 in the 14–15 year group (n = 2) (Fig 2A). The pulp/tooth ratio of the 14–15 year group was significantly lower than that of the 2–4, 5–7, and 8–10 year groups in primary teeth among healthy participants. The pulp/tooth ratio of permanent teeth in healthy participants was 22.8 in the 2–4 year group (n = 1), 21.4 ± 0.1 in the 5–7 year group (n = 2), 17.1 ± 0.4 in the 8–10 year group (n = 41), 17.1 ± 0.4 in the 11–13 year group (n = 55), and 16.1 ± 0.3 in the 14–15 year group (n = 57) (Fig 2B). The pulp/tooth ratio of the 8–10 year group was significantly higher than that of the 11–13 and 14–15 year groups in permanent teeth among healthy participants.

**Table 2:** Number of analyzed teeth in healthy participants and X-linked hypophosphatemia (XLH) patients.

	Second primary molar		First molar	
	Healthy	XLH	Healthy	XLH
2–4 years	47	12	0	0
5–7 years	35	20	2	0
8–10 years	10	2	41	13
11–13 years	1	0	55	18
14, 15 years	2	0	57	0
Total	95	34	155	31

**Fig 3:**
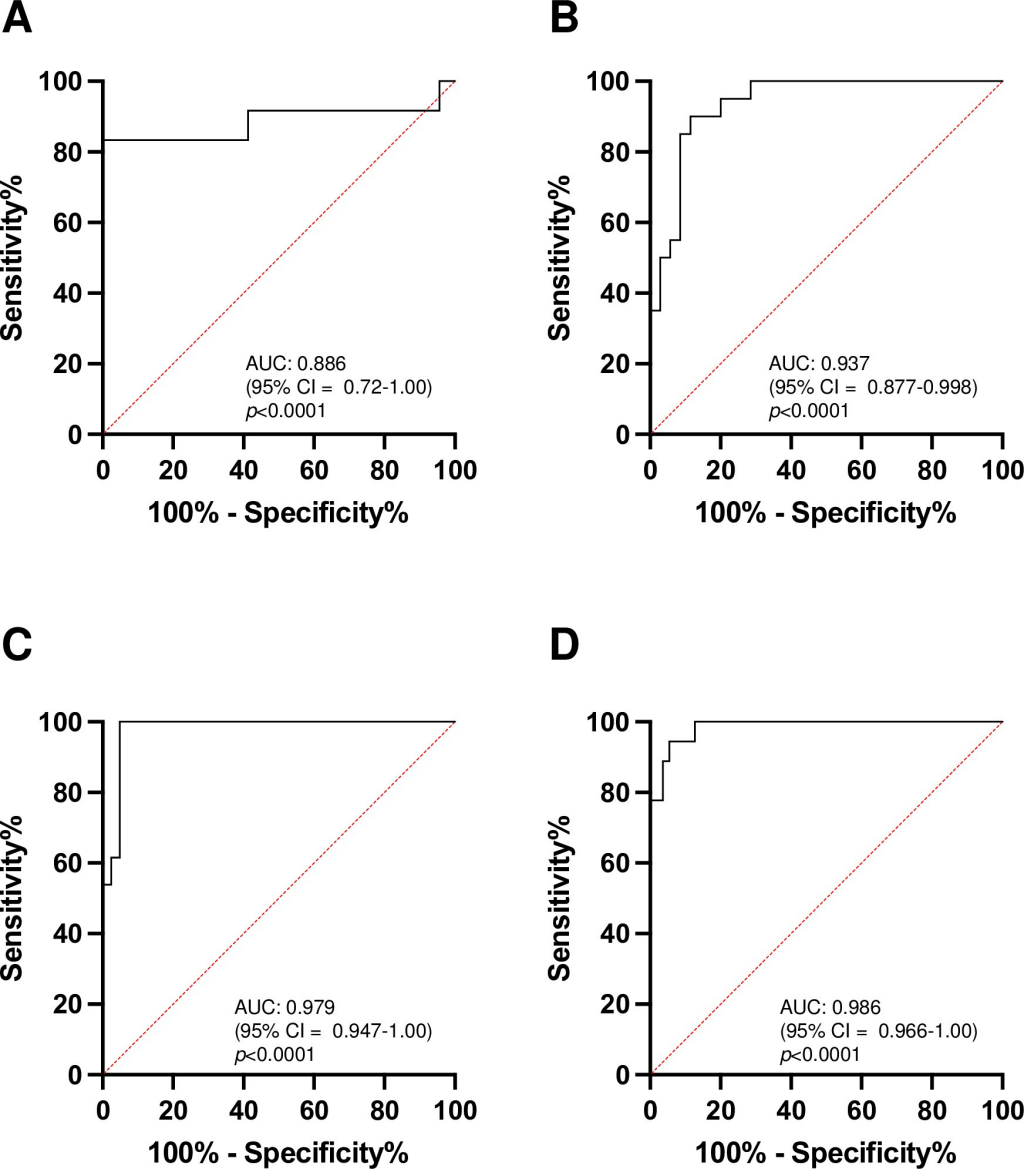
Receiver operating characteristic curves of pulp/tooth ratio predicting dentin dysplasia of X-linked hypophosphatemia (XLH). (A) Second primary molar in 2–4 year age group. (B) Second primary molar in 5–7 year age group. (C) First permanent molar in 8–10 year age group. (D) First permanent molar in 11–13 year age group.

The ROC curves of the pulp/tooth ratio are presented in Fig 3. The diagnostic accuracy of the pulp/tooth ratio for XLH was high, with an AUC of 0.886 in the 5–7 year group, 0.937 in the 8–10 year group, 0.979 in the 11–13 year group, and 0.986 in the 14–15 year group. The cutoff score for the pulp/tooth ratio that best differentiated individuals with XLH was 27.9 (sensitivity 83.3% and specificity 100%) in the 5–7 year group, 24.7 (sensitivity 90.0% and specificity 88.6%) in the 8–10 year group, 24.9 (sensitivity 100% and specificity 95.1%) in the 11–13 year group, and 27.9 (sensitivity 94.4% and specificity 94.6%) in the 14–15 year group.

**Fig 4:**
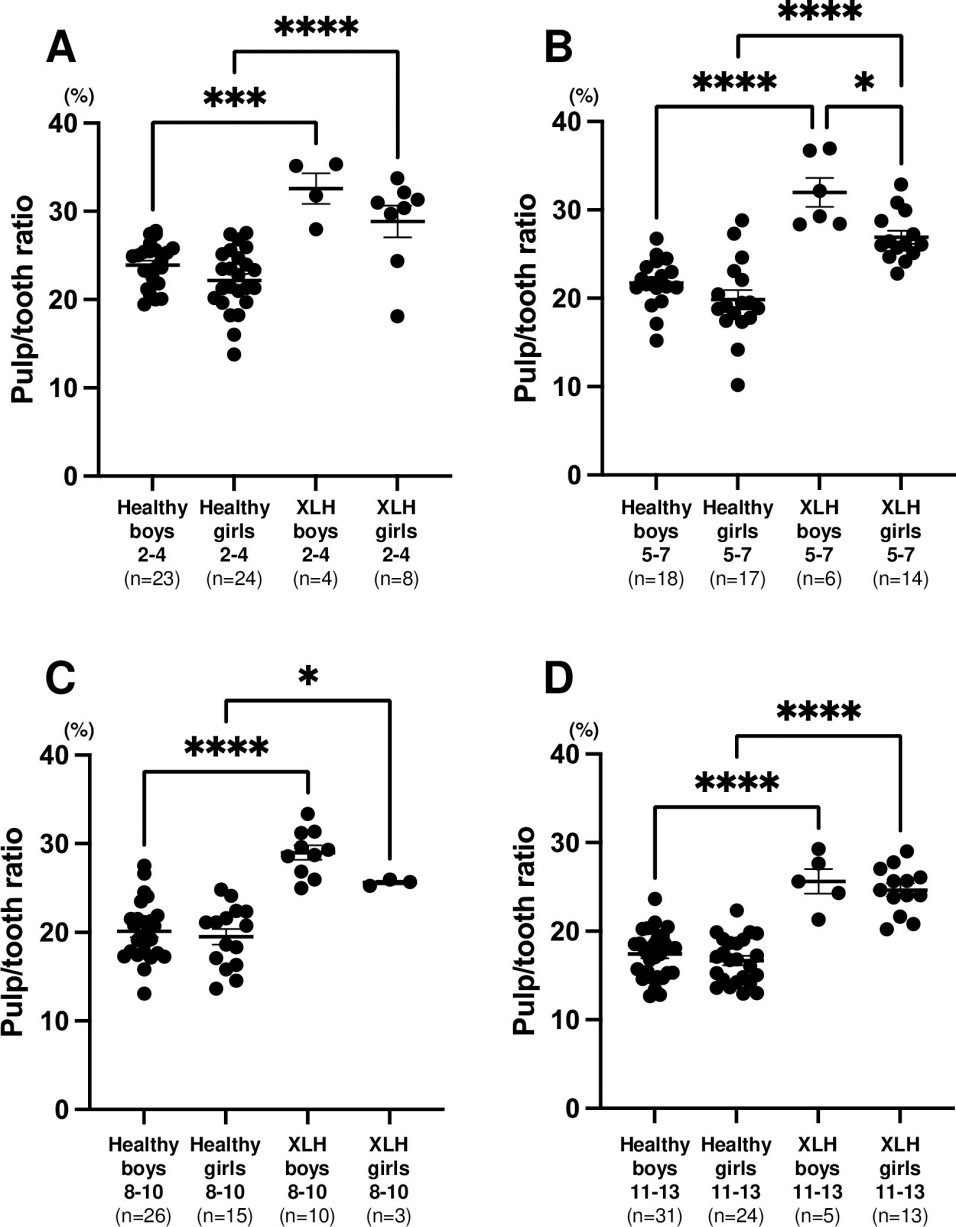
Comparison of pulp/tooth ratio of patients with X-linked hypophosphatemia (XLH) and healthy participants by sex. Black horizontal lines in each age group indicate average ± SEM. *p < 0.05, ***p < 0.001, and ****p < 0.0001 between groups. (A) Second primary molar in 2–4 year age group. (B) Second primary molar in 5–7 year age group. (C) First permanent molar in 8–10 year age group. (D) First permanent molar in 11–13 year age group.

In patients with XLH, significantly higher values were observed in second primary molars compared with the values in healthy children aged 2–4 (boys: *p* < 0.01, girls *p* < 0.001) and 5–7 years (*p* < 0.001) (Fig 4A and 4B). Additionally, the values for the first permanent molars were significantly higher than those of healthy children aged 8–10 (boys: *p* < 0.001 vs girls *p* < 0.05) and 11–13 (*p* < 0.001) (Fig 4C and 4D). No difference was found between boys and girls in pulp/tooth ratio except in second primary molars in XLH children aged 5–7 years (*p* < 0.05) (Fig 4B).

### Correlation of pulp/tooth ratio and dental abscesses or treatment in patients with XLH

**Fig 5:**
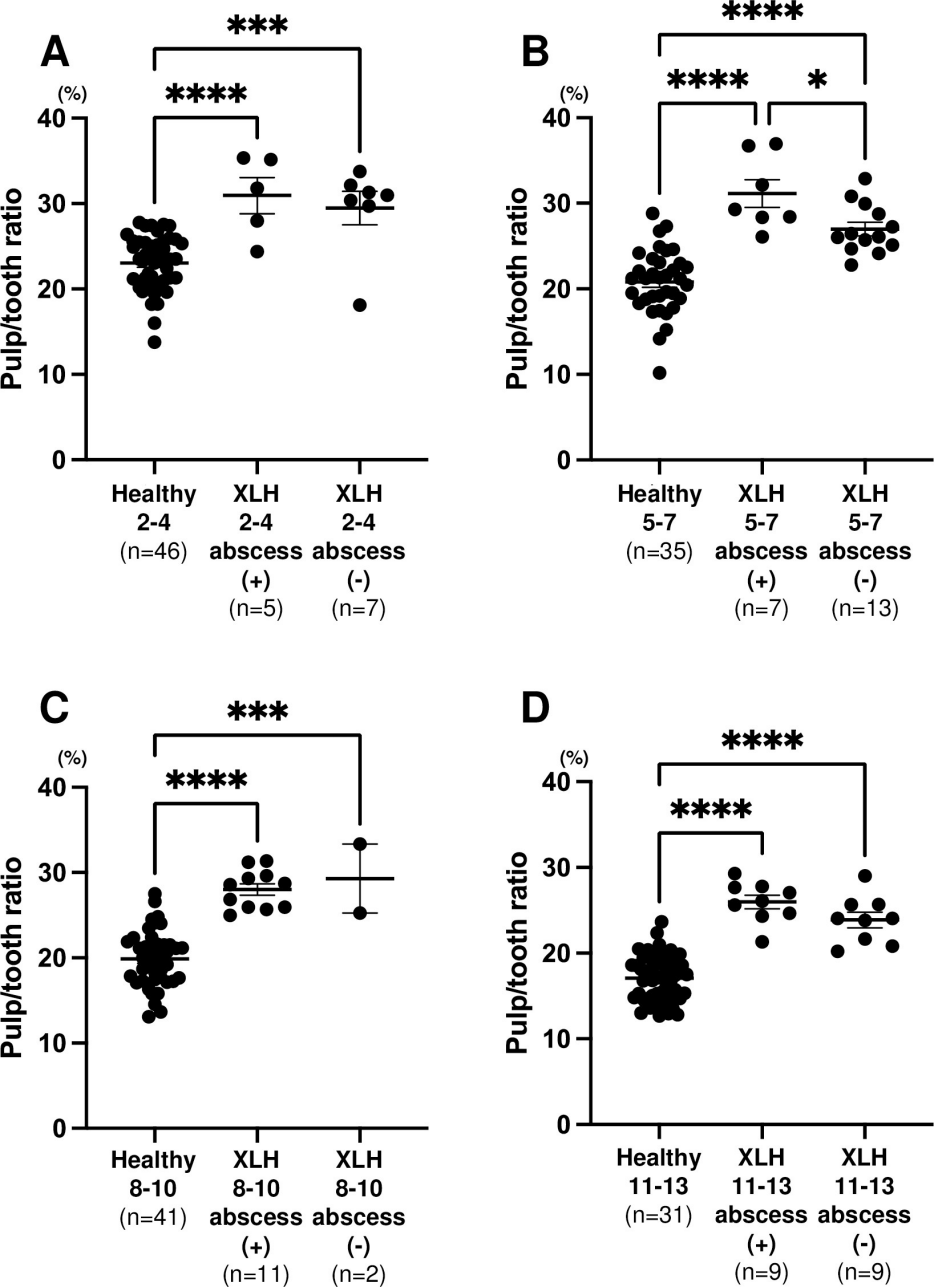
Comparison of pulp/tooth ratio of patients with X-linked hypophosphatemia (XLH) with or without experience of dental abscesses and healthy participants. Black horizontal lines in each age group indicate average ± SEM. *p < 0.05, ***p < 0.001, and ****p < 0.0001 between groups. (A) Second primary molar in 2–4 year age group. (B) Second primary molar in 5–7 year age group. (C) First permanent molar in 8–10 year age group. (D) First permanent molar in 11–13 year age group.

**Fig 6:**
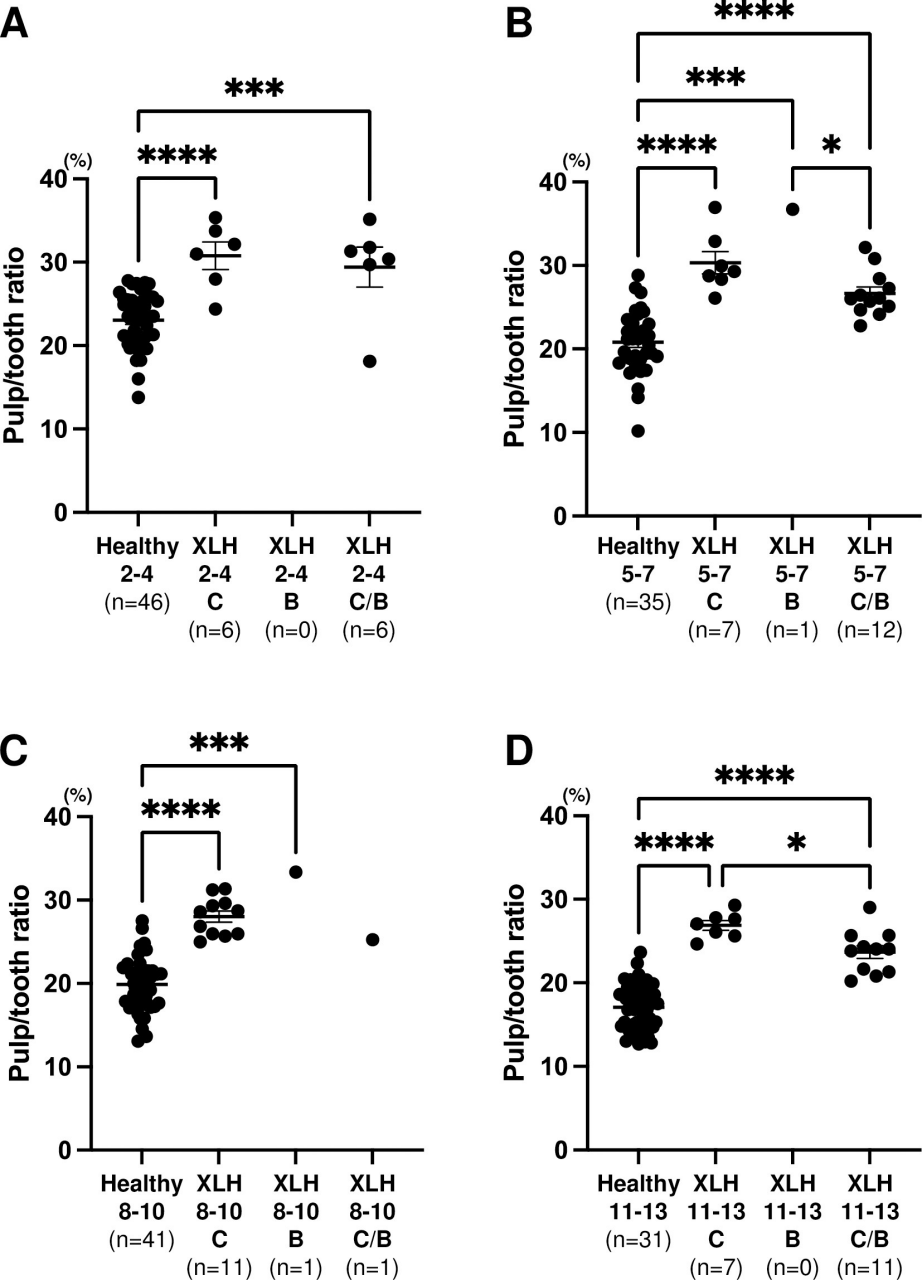
Comparison of pulp/tooth ratio of patients with X-linked hypophosphatemia (XLH) by treatment method. Black horizontal lines in each age group indicate average ± SEM. *p < 0.05, **p < 0.01, ***p < 0.001, and ****p < 0.0001 between groups. (A) Second primary molar in 2–4 year age group. (B) Second primary molar in 5–7 year age group. (C) First permanent molar in 8–10 year age group. (D) First permanent molar in 11–13 year age group. C: conventional therapy. B: burosumab. C/B: transferred therapy from conventional to burosumab.

Fig 5 shows that no significant difference in pulp/tooth ratio was observed between the XLH patients with and without dental abscesses (*p* = 0.6324: 2–4 years, *p* = 0.5573: 8–10 years, *p* = 0.1038: 11–13 years), except in XLH patients aged 5–7 years (*p* = 0.0180). No difference was found between conventional therapy and burosumab therapy in pulp/tooth ratio; however, the pulp/tooth ratio of the transferred group was lower than that of the burosumab group in the second primary molar of XLH patients aged 5–7 years and the conventional therapy group in the first permanent molar of XLH patients aged 11–13 years (Fig 6).

## Discussion

### Establishment of a quantitative method for evaluating dentin dysplasia using orthopantomography

Dentin dysplasia of XLH patients is evident clinically [5–7]. A large pulp chamber, thin dentin, and prominent pulp horns are seen radiographically as manifestations of dentin dysplasia [5, 6, 8]. The purpose of this study was to determine whether the severity of dentin dysplasia could be quantitatively evaluated by measuring the area of the pulp cavity in XLH patients, and to establish reference values for healthy children of the same age. In the healthy group, the pulp chamber became narrower with increasing age. This reflects the formation of secondary dentin in response to stimulation after eruption [21]. We chose to analyze mandibular molars rather than maxillary molars. The roots of maxillary primary molars are difficult to analyze because of the overlapping of primary molars and the tooth germs of permanent successors. The roots of the maxillary permanent molars are also difficult to analyze because of overlapping of the floor of the palatal cavity. The second primary molar and the first permanent molar were selected as the target teeth. The root of the second primary molar is completely formed at the age of 3 years, and root resorption by the permanent successor starts at the age of 8 years [22]. The root of the first permanent molar is completely formed at the age of 8 years [23]. This study revealed that this standard value of the second primary molar can be used as an index for ages 2–7, and the value of the first permanent molar can be used as an index for ages 8 years and older. A previous report determined the size of the pulp chamber compared with age-matched healthy participants aged approximately 6 years [24]. Our study made it possible to evaluate the pulp cavity area from the primary dentition and to diagnose the need for early intervention. A limitation of this study was the difficulty in distinguishing minor dental caries, slight attrition, or worn teeth. This limitation was minimized as much as possible by having one trained pediatric dentist analyze a large number of teeth.

### Evaluation of dentin dysplasia in XLH patients

One report of quantitative analysis using high‐resolution μCT and the histology of extracted primary teeth of patients with XLH revealed dental mineralization defects associated with XLH [25]. However, in clinical practice, it is important to diagnose the existing teeth in the oral cavity. No practical methods are available for diagnosing dentin dysplasia in XLH patients. This study established optimal cutoff pulp/tooth ratios for predicting dentin dysplasia in XLH patients. These results provide objective, practical, and useful indications for diagnosing the severity of dentin dysplasia in XLH patients. In this study, the pulp/tooth ratio was higher in XLH patients than in healthy participants, indicating that XLH patients had large pulp chambers caused by dentin dysplasia.

The different expression of XLH between male and female individuals has not been fully confirmed. Some studies have suggested that XLH is an X-linked dominant disorder in which the symptoms are mediated by lyonization [5, 26]. Dental and bone manifestations tend to be more severe in males than in females. In this study, dental abscesses in the primary dentition were detected significantly more often in boys than girls, and the pulp chambers of boys tended to be wider than those of girls, indicating that dentin dysplasia in XLH is more severe in boys than in girls. Dental abscesses sometimes lead to a diagnosis of XLH [8, 9]. Dentists should refer patients to a pediatrician as soon as possible if they find a dental abscess in the absence of dental caries or dental trauma, especially in boys. Early diagnosis of XLH enables not only early management of growth and development by a pediatrician, but also oral management from the primary dentition to the permanent dentition, with a particular focus on preserving the pulp. Preservation of the pulp allows normal replacement of primary teeth and extends the life of the permanent tooth.

Although there was no significant difference in the width of the pulp chamber between XLH patients with or without experience of dental abscesses except for second primary molars in the 5–7 year age group, XLH patients who experienced dental abscesses tended to have larger pulp chambers than XLH patients who did not experience dental abscesses. If dentists diagnose dentin dysplasia based on a large pulp chamber before dental abscess formation, aggressive treatment such as coronal restorations in the primary and permanent dentition can be commenced to prevent pulp infection [7, 10]. Additionally, large pulp chambers should be considered during tooth preparation to prevent the pulp from being exposed or irritated. Restoration methods that require extensive tooth reduction should be avoided [5, 8].

There was also no significant difference in the width of the pulp chamber among XLH patients who were treated with only conventional therapy, with burosumab alone, and with conventional therapy followed by burosumab; however, the transferred group had narrower pulp chambers than the burosumab group in the 5–7 year age group and the conventional therapy group in the 11–13 year age group. This result indicates that both treatments for bone do not fully restore the hypomineralization of the teeth to the same level as that of healthy children. Dentin does not undergo any remodeling, nor does it participate in the regulation of calcium and phosphate metabolism [27]. Mineralization of the second primary molar starts at around the sixth month of pregnancy, and root formation is complete by the age of 3 years. Mineralization of the first permanent molar starts at birth, and root formation is complete by the age of 9 years. The first permanent molar might be suitable for long-term monitoring of the dental effects of conventional therapy or burosumab on dentin. Treatment with burosumab has only been recently introduced [28], and its effectiveness may be revealed when long-term cases increase in number.

A major limitation of this study is the small number of patients with XLH because of the rarity of this disease. Only 17 patients with XLH were included in this study, which was not sufficient for statistical analysis. Another limitation was that only retrospective data were available, and medical information such as date of diagnosis and start of medical treatment was not available. Further research collaborating with medical and dental region should be undertaken with a greater number of XLH cases including medical information. However, a strength of this study was the use of orthopantomography, a commonly used imaging modality in dental practice that serves as a valuable diagnostic tool. Our new methods have broad utility given that orthopantomography is available in most dental clinics. In future research, orthopantomographic images of patients aged over the age of 15 years should be collected to enable further long-term longitudinal evaluation using first permanent molars.

In conclusion, we established a novel methodology for using orthopantomography to quantitatively evaluate dentin dysplasia from the primary dentition through to the permanent dentition. Evaluation of the severity of dentin hypomineralization using this method is a useful tool in the diagnosis of the dental manifestations of XLH.

## Supporting information

S1 Data (XLSX)
